# Orthodontic treatment in patient with idiopathic root resorption: A case report

**DOI:** 10.1590/2176-9451.20.1.108-117.oar

**Published:** 2015

**Authors:** Diego Rey, Rosana Martínez Smit, Liliana Gamboa

**Affiliations:** 1 Assistant Professor and Head, Department of Orthodontics, CES University, Medellín, Colombia; 2 Assistant Professor, Department of Orthodontics, CES University, Medellín, Colombia; 3 Specialist in Orthodontics

**Keywords:** Root resorption, Orthodontics, Corrective Orthodontics, Tooth resorption

## Abstract

Multiple idiopathic external root resorption is a rare pathological condition usually detected as an incidental radiographic finding. External root resorption of permanent teeth is a multifactorial process related to several local and systemic factors. If an etiological factor cannot be identified for root resorption, the term "idiopathic" is applied. This report presents a case of multiple idiopathic apical root resorption. The condition was found in a young female patient seeking orthodontic treatment due to malocclusion. This kind of resorption starts apically and progresses coronally, causing a gradual shortening and rounding of the remaining root. Patients with this condition are not the ideal candidates for orthodontic treatment; however, the aim of this report is to describe an unusual case of idiopathic root resorption involving the entire dentition, and to present the orthodontic treatment of this patient. It describes the progress and completion of orthodontic therapy with satisfactory end results.

## Abstract (pt)

A reabsorção radicular externa idiopática é uma rara condição patológica, normalmente detectada como um achado fortuito radiológico. Trata-se de um processo multifatorial, relacionado a diversos fatores locais e sistêmicos. Se um fator etiológico não for identificado, a reabsorção radicular é classificada como idiopática. Apresentamos o caso de uma paciente do sexo feminino com reabsorção radicular idiopática, que procurou tratamento ortodôntico para corrigir uma má oclusão. Esse tipo de reabsorção começa apicalmente e evolui coronalmente, provocando o encurtamento gradual e o arredondamento da raiz remanescente. Os pacientes com essa condição não são os candidatos ideais para o tratamento ortodôntico, no entanto, o objetivo desse relato é descrever um caso incomum de reabsorção radicular idiopática que envolveu toda a dentição. Além disso, pretende-se apresentar o tratamento ortodôntico da paciente. Também são descritos o progresso e a conclusão do tratamento ortodôntico, assim como os satisfatórios resultados finais.

## INTRODUCTION

External root resorption in the permanent dentition is usually pathological. Recognized causes of external resorption of primary and permanent teeth include trauma, infection, periodontal disease, endodontic treatment, encroachment from neoplasm, orthodontic treatment, bleaching, Paget's disease of bone, and trauma to the jaws. When none of these causes are present, resorption is termed ''idiopathic resorption of teeth.''^1^ Idiopathic external root resorption (IERR) affects either or both apical and cervical regions of one or several teeth, but most commonly occurs in the apical region. It is relatively rare to find idiopathic resorption in the cervical areas of a tooth, and even more uncommon for the condition to involve multiple teeth.^2^

The first report was published in 1930,^3^ and described a case of progressive cervical root resorption associated with functional hepatic disturbances.

The incidence of IERR seems to be greater in younger women.^4^^,^ 5 Only nine clearly identified cases of multiple idiopathic apical root resorption have been reported in the literature.^1^^,^ 3 ^,^ 6 ^-^ 12 All of them were in relatively young individuals aged from 14 to 34 years old, and all except two were in males.^11^^,^ 12

This type of root resorption might have a hereditary familiar component, and can be detected in siblings of a similar age.^13^ It is also related to other dental anomalies, as early loss of primary teeth, agenesis, invaginated teeth, conoid teeth, supernumerary teeth, microdontia, taurodontia and pulp calculus.^13^^-^ 16 Also, it can be associated with syndromes such as Down and Steven Johnson.^17^ The clinical shape of IERR does not differ from those of known etiology. Although external root resorption is most commonly diagnosed by evaluation of radiographs,^18^ the diagnosis of IERR must be an exclusion of local factors and medical conditions and, therefore, the medical history of the patient plays an important role when there is no evidence of an etiological triggering factor.^19^

IERR presents a common group of characteristics that include involvement of several or all teeth, clinically asymptomatic, which respond to pulp vitality tests and might present mobility, decreased alveolar bone and poor periodontal insertion.^10^ Radiographic resorption begins at the cement-enamel junction or in the apical area and there is a loss of more than one third of root length. Histological tests of removed soft tissue of teeth reveal non-specific chronic inflammation.^10^

No reports were found in the literature regarding orthodontic management of patients with multiple idiopathic root resorption and which document long term post-treatment stability and prognosis. This article describes a case of severe idiopathic apical root resorption in which no cause could be identified or any reason determined for its occurrence. Also, orthodontic management aimed at solving the esthetic and functional concerns of the patient. This research also describes the clinical and radiographic findings, as well as the biomechanical management during the evolution of treatment.

## DIAGNOSIS AND ETIOLOGY

A 17-year-old female patient whose chief complaint was the presence of diastemas in the maxillary anterior region, an esthetic and psychological concern that she described inhibited and limited her interaction with other people, presented for treatment. She was also concerned about the potential risk of losing some of her teeth due to general root resorption which had been previously diagnosed by another orthodontist who had refused to treat her due to the potential risks involved in trying to close the spaces.

**Figure 1 -:**
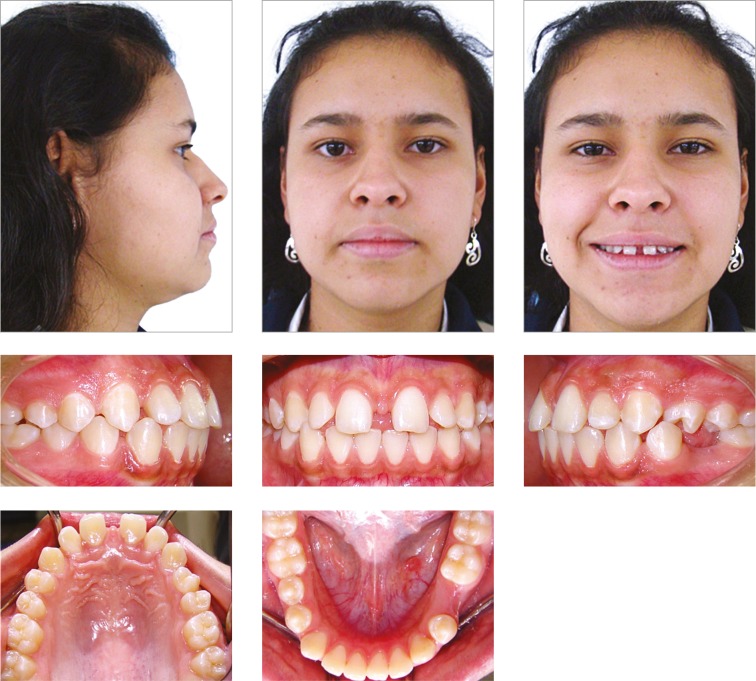
Initial facial and intraoral photographs.

The patient presented a straight profile, good health condition and oral hygiene, normal breathing pattern and atypical swallowing pattern (Fig 1). Intraoral examination revealed Class I malocclusion, 2-mm overjet and 5% overbite, coinciding dental midlines, moderate spacing in both arches and upper and lower labialized and protruded incisors (Figs 1 and2). Radiographic analysis revealed the presence of all teeth which exhibited altered crown-root proportion, (maxillary right permanent lateral incisor, mandibular right first and second premolars) with thinned and short roots, sclerosis of root canals and complete root resorption of maxillary permanent left lateral incisor. Tooth buds of maxillary and mandibular left third molars at Nolla Stage 6 development were observed, as well as the presence of mandibular second primary molar with congenital absence of mandibular left second premolar and mandibular right third molar (Fig 3). The patient presented Class I skeletal pattern with bimaxillary prognathism and macrognathism, proclination of maxillary and mandibular incisors and acute nasolabial angle (T_0_) (Fig 3 andTable 1).

**Figure 2 -:**
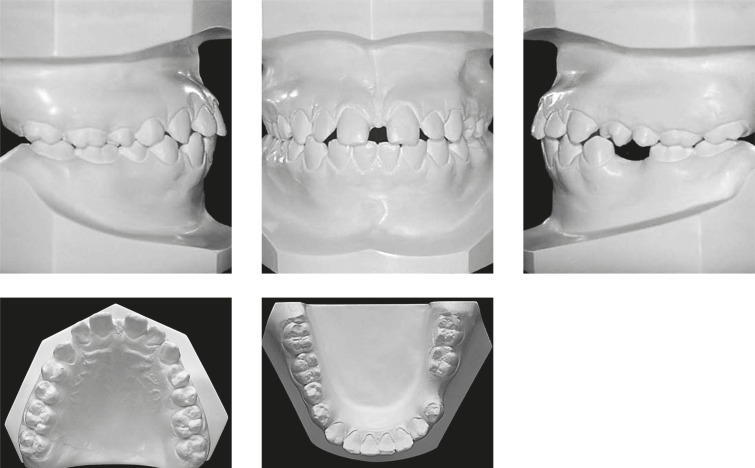
Initial casts.

**Figure 3 -:**
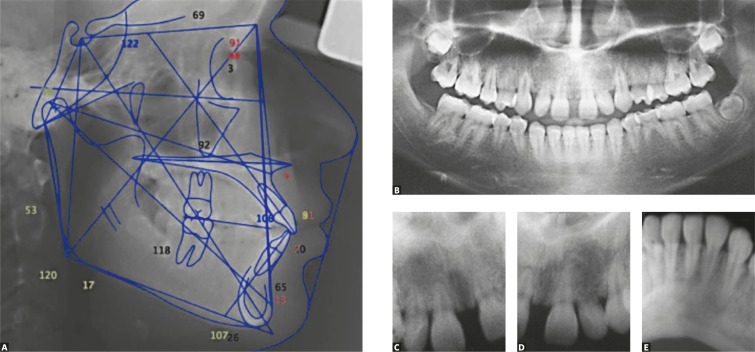
Initial radiographs. A) Cephalometric tracing; B) Panoramic radiograph; C) Periapical radiograph of right upper incisors; D) Periapical radiograph of left upper incisors; E) Periapical radiograph of lower incisors.

**Table 1 -:** Cephalometric measurements

Measurement	Norm	T_0_	T_1_	T_1_-T_0_
SNA (degrees)	76.2 - 83.8	91.3	89.8	-1.5
SNB (degrees)	75 - 81	88.5	86.8	-1.7
ANB (degrees)	5.1 - 0.5	2.8	3	0.2
Co-A (mm)	90	91.7	92.1	-0.4
Co-Pog (mm)	110	118.1	117.9	-0.2
FMA (degrees)	24.2	18.2	18.8	0.6
Nasolabial angle (degrees)	105	91.2	97.1	5.9
Lower lip to H line (degrees)	0 - 0.5	3	0	-3
U1-FH (degrees)	110	129.9	113.3	-16.6
U1-PP (degrees)	105 - 115	128.5	115.7	-12.8
L1-PM (degrees)	88.5 - 97	106.6	102.8	-3.8
Interincisal angle (degrees)	124	106.5	127.5	-21

There was no previous history of orthodontic treatment, all teeth presented normal response to electrical and heat pulp tests and were negative upon percussion and palpation. Sporadic painful symptomatology of posterior segments was reported during mastication. All teeth presented normal physiological mobility, except for maxillary left permanent lateral incisor that had grade II mobility. Anatomy and color of crowns were normal. Periodontal examination indicated normal probing depths between 2 and 3 mm without bleeding.

## TREATMENT OBJECTIVES

The aim of orthodontic treatment was mainly to meet patient's esthetic expectations, achieve closure of anterior diastemas with light forces and also maintenance of crown-root proportion.

## TREATMENT ALTERNATIVES

Treatment options for this patient were limited due to her dental characteristics and malocclusion. At first, orthodontic treatment was not an option, but the patient was highly concerned about esthetics. Another option was not using Orthodontics to fully close diastemas between maxillary teeth, but distributing those spaces to be restored with composites instead, so as to increase mesiodistal width, and also restore with osseointegrated implants the absent premolar and maxillary permanent left lateral incisor. Nevertheless, the patient did not count with the economic resources for this treatment option. Thus, it was decided to start orthodontic treatment focused on fully closing diastemas with light forces. The patient agreed and understood the risks.

## TREATMENT PROGRESS

Prior to treatment onset, the patient was informed about the characteristics of the progressive pulp pathology condition she had and the limitations, risks and objectives of treatment. After signing an informed consent form, orthodontic therapy was initiated.

Treatment plan required initial consultation with an endodontist in order to evaluate the degree and severity of external root resorption and begin orthodontic treatment with minimal risk, while taking into account the existing limitations.

**Figure 4 -:**
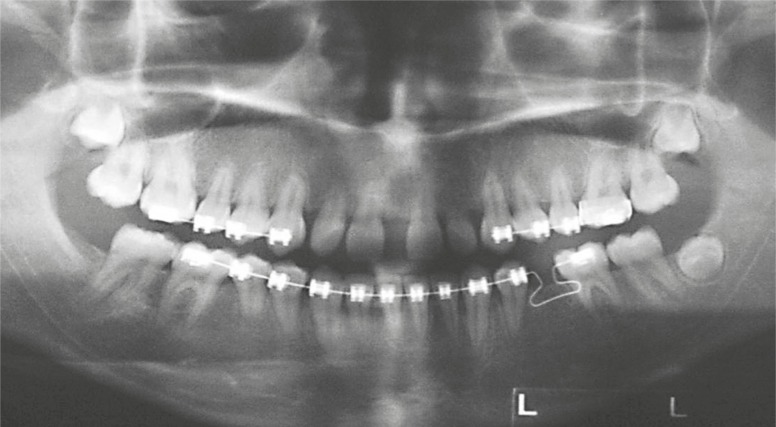
Control panoramic radiograph.

**Figure 5 -:**
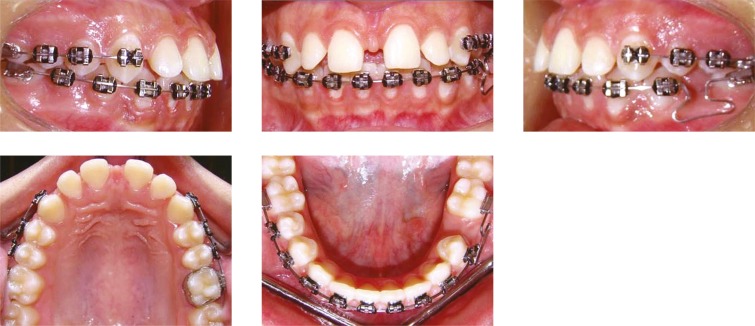
Intraoral photographs during orthodontic treatment.

Orthodontic treatment initiated first in the upper posterior segments between canines and molars with an edgewise-standard technique. During the first phase of treatment, low caliber NiTi wires were used (Fig 4). Once the posterior segments of the maxillary arch were consolidated, fixed appliances were installed in the upper anterior segment where teeth were more affected by resorption. Space closure in the lower arch was initiated with a frictional technique using light elastomeric chains. Strict panoramic radiographic control was carried out every eight months based on clinical criteria in order to monitor the progression of pulp pathology (Fig 5). Given the positive response during treatment, the space between mandibular first premolar and molar was closed by attraction with a closed loop which had a tip back bend on the molar in order to protract and disincline it (Fig 4).

Esthetic contouring of upper anterior crowns was not necessary given the fact that all spaces were closed satisfactorily, thereby achieving an adequate distribution of all spaces. Prosthetic replacement of the maxillary lateral incisor was also not necessary due to stability shown during treatment. During the final phase of treatment, the patient was referred to maxillary labial frenectomy and speech therapy in order to control tongue thrust habit that could affect long-term stability of treatment. Retention was completed with maxillary and mandibular fixed retainers from canine to canine and the use of ESSIX plates. Total treatment time was 2.3 years between 2009 and 2011. Some treatment limitations were encountered during the final phase of treatment, such as the impossibility of completely aligning midlines due to the initial absence of mandibular left first molar. The spaces between mandibular second premolar and mandibular second molar were also not closed completely due to occlusal adjustment in that segment, which required tip-back biomechanical movements that represented high risk of root resorption. Post-treatment periodic radiographic controls were recommended to monitor the progression of root resorption.

## TREATMENT RESULTS

**Figure 6 -:**
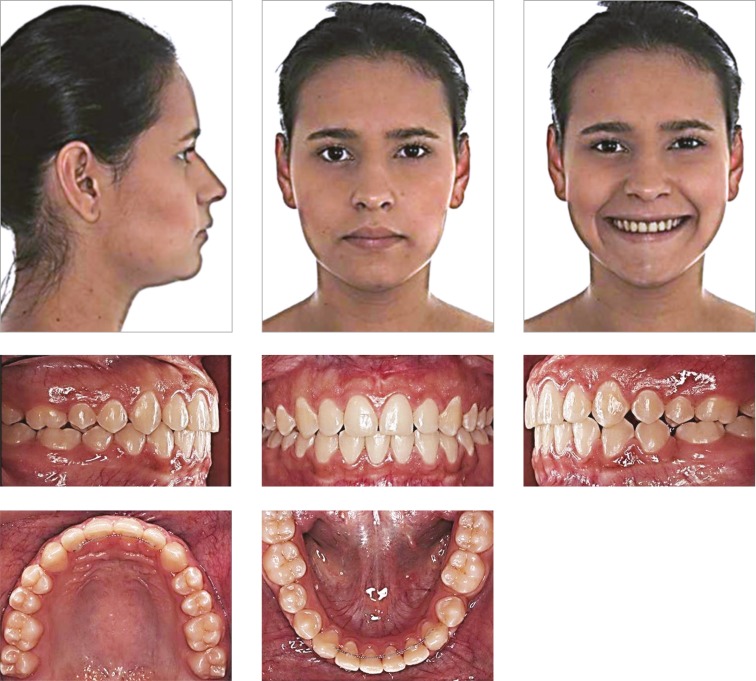
Post-treatment facial and intraoral photographs.

**Figure 7 -:**
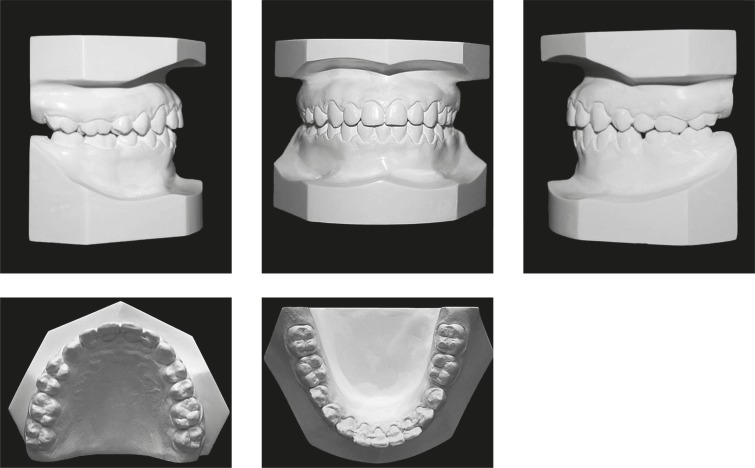
Post-treatment casts.

After orthodontic treatment with fixed appliances, the shape and contour of both dental arches improved, the rotations were fixed, diastemas were closed, proclination of maxillary and mandibular incisors was improved, a better occlusal relationship was achieved, overbite and overjet were corrected, the Curve of Spee was flattened, her nasolabial angle improved (T_1_), and a harmonic smile was achieved (Figs 6,7 and8).

**Figure 8 -:**
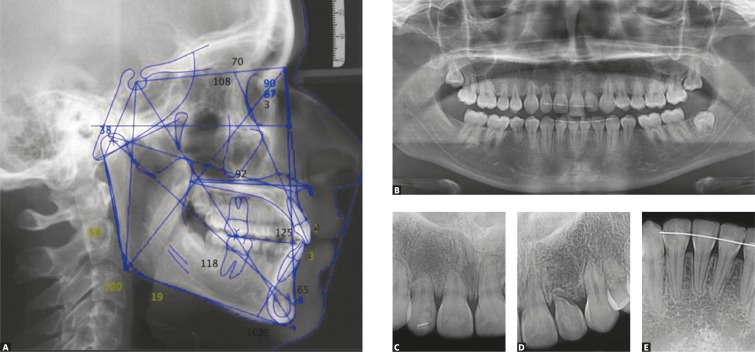
Post-treatment radiographs. A) Cephalometric tracing; B) Panoramic radiograph; C) Periapical radiograph of right upper incisors; D) Periapical radiograph of left upper incisors; E) Periapical radiograph of lower incisors.

Panoramic and periapical radiographs taken at the end of treatment revealed that there was no significant progression of root resorption and the periodontal condition was acceptable (Fig 8).

## DISCUSSION

Clinical reports of classical idiopathic multiple root resorption are presented for patients whose past medical history did not reveal any associated systemic, dental or familial causes.^1^^,^ 3 ^,^ 6 ^-^ 12 This article presented the orthodontic management of a young female patient with severe root resorption whose teeth were preserved esthetically and functionally. It is important that the clinician have an understanding of the incidence, cause and effects of root resorption in order to offer patients the best treatment options.

The literature suggests that two types of idiopathic root resorption have been observed: apical and cervical. Cervical root resorption starts in the cervical area of teeth and progresses towards the pulp. In the apical type, resorption starts apically and progresses coronally, causing a gradual shortening and rounding of the remaining root.^20^

In this report, a patient with apical external root resorption with gradual rounding and shortening of roots was orthodontically treated. It was possible to reduce protrusion on both sides by decreasing U1-FH -16^o^, U1-PP -12.8^o^ and L1- MP -3.8^o^ (Table 1). The nasolabial angle reduced in 5.9^o^ while the lower lip retruded 3 mm (Table 1), thereby improving patient's profile (Fig 6). The condition remained stable during the course of orthodontic treatment.

Marques et al^21^ reported a case of a young girl diagnosed with a condition described as short root anomaly (SRA), a pathology similar to IERR described on this paper; however, SRA is established when family link is established. The authors highlighted the importance of good diagnosis and effectiveness of orthodontic therapy that did not involve force applied directly on affected teeth.

The origin of the condition does not seem to be in the pulp and, therefore, interceptive endodontic treatment that includes pulp removal and placement of calcium hydroxide or gutta-percha are not indicated.^22^

Given that dental and bone resorption is caused by osteoclastic activity,^22^ it is hypothesized that there is some triggering factor that activates these cells.

Current management of this condition is conservative, minimally invasive and consists of long-term monitoring.^23^ Orthodontic treatment is a viable alternative that offers patients an acceptable esthetic and functional solution. However, there are important considerations that the orthodontist must take into account and follow, such as the prognosis of teeth with a history of severe resorption, progression of the condition, progress and stability of teeth with future restorations.

These cases are best described as idiopathic because no cause or family history could be associated. Management of interceptive therapy of idiopathic root resorption depends on the identification of the specific cell mechanism and the external factor that cause the disorder. Orthodontic management is an useful alternative that provides these patients with a functional and esthetic option. It is important that the clinician completes a full medical history and detailed initial clinical and radiographic findings and have the patient sign an informed consent document prior to treatment onset. Orthodontic therapy should be focused on solving patient's esthetic concerns.

Oyana et al,^24^ using the finite element method, demonstrated that a significant amount of stress was concentrated at the middle of the root in a model of short root. That condition is sufficient to increase root resorption in progress on those patients. Orthodontic forces should be applied with caution. In alignment and leveling, the use of intermittent, light and constant forces that do not surpass capillary blood pressure of 20-26 g/cm^2^ are recommended. The use of Class II intermaxillary elastics, maxillary expansion appliances anchored on premolars and extraoral forces anchored on first molars should be avoided, since they have been reported as a potential risk factor for teeth with root resorption.^26^

Strict radiographic controls during the course of orthodontic therapy in order to monitor the resorptive condition are very important. During retention, fixed retainers in the upper and lower anterior segments are recommended. It is also important to identify the presence of functional habits, such as atypical swallowing or nail biting, both of which could affect treatment results and stability of compromised teeth.

It is also important to emphasize the need to insist on extreme oral hygiene measures in order to maintain patient's periodontal stability. Post-treatment radiographic control is recommended in order monitor the condition and establish a long-term prognosis, in addition to addressing the concerns mentioned above.

## CONCLUSIONS

1) Orthodontic treatment of patients with idiopathic multiple root resorption offering them esthetical and physiological solutions is possible considering that the patient understands potential risks and limitations.

2) Orthodontic management is based on simple mechanical techniques that include light and controlled forces, allowing predictable movements which are physiologically acceptable if pulp and periodontal limitations are considered.

3) A complete history of patient's medical background allows identification of any systemic condition that might be associated with the pulp pathology.

4) An informed consent form is indispensable and protects the clinician in case of any legal implication that might arise in these types of cases.
